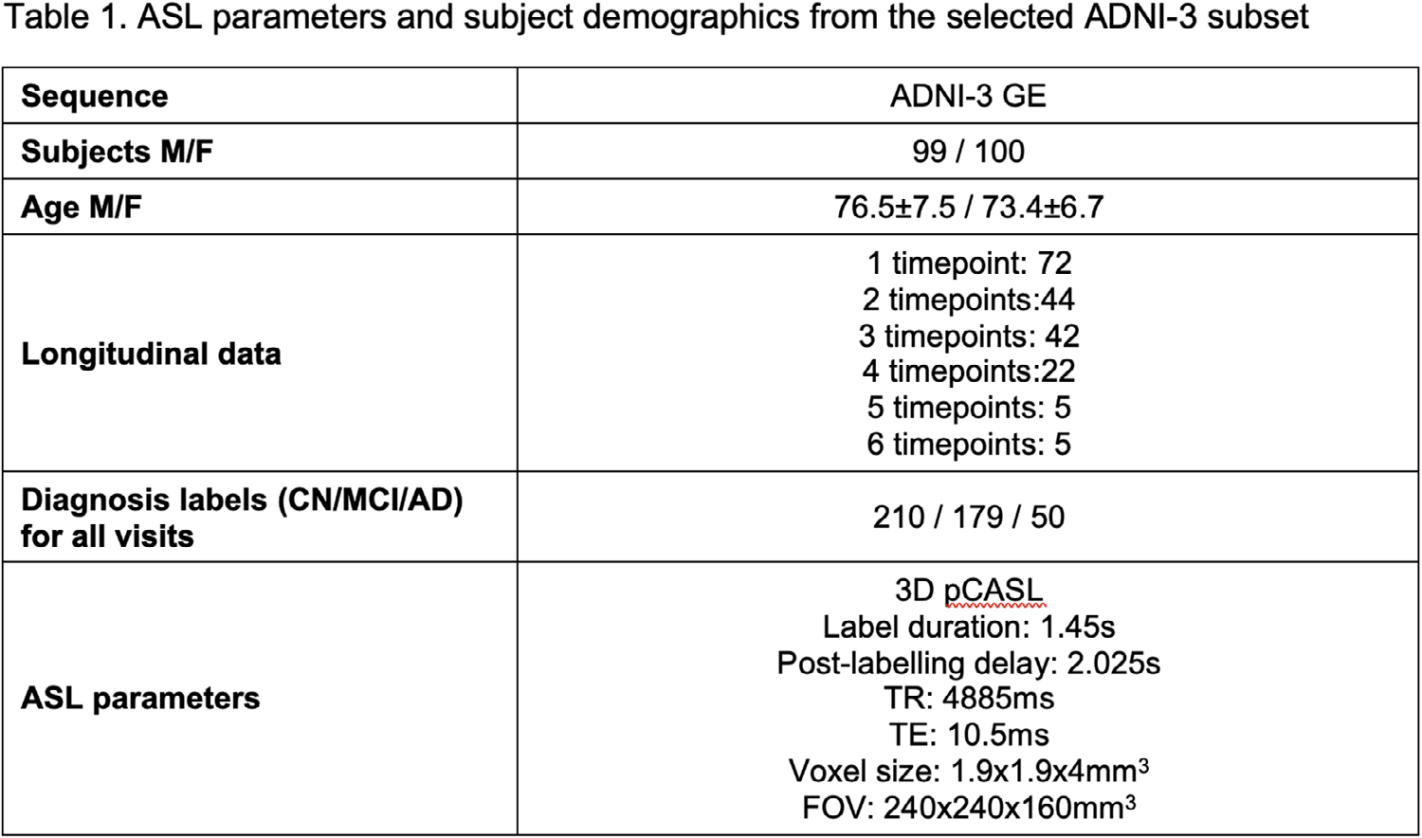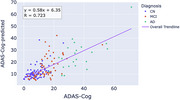# Correlation between cognitive scores in Alzheimer's disease and cerebral perfusion measured by arterial spin labelling MRI

**DOI:** 10.1002/alz70862_109937

**Published:** 2025-12-23

**Authors:** Avital Dell'Ariccia, Armen Bodossian, Logan Xin Zhang, Thomas Kirk, Martin Craig, Michael Chappell

**Affiliations:** ^1^ Quantified Imaging, London, London UK; ^2^ University of Nottingham, Nottingham, Nottinghamshire UK

## Abstract

**Background:**

Arterial Spin Labelling (ASL) is a non‐invasive MRI technique that enables the measurement of cerebral blood flow (CBF), which is known to change throughout Alzheimer’s disease (AD) progression^1^. CBF within a defined brain region of interest (ROI), or multiple regions, might be used as an imaging biomarker in dementia. This study introduces a novel AD meta‐ROI derived from ASL data and assesses its correlation with cognitive scores to investigate whether ASL could serve as an alternative to PET and other invasive biomarkers.

The objective was to track disease progression across the cognitively normal (CN), mild cognitive impairment (MCI), and AD stages.

**Method:**

We analysed a subset of subjects from Alzheimer’s Disease Neuroimaging Initiative (ADNI3) (Table 1). ASL data was processed using *qasl* pipeline (Quantified Imaging, London, UK), which follows the principles outlined by Alsop^2^ using a Bayesian algorithm^3^ to obtain calibrated CBF maps, normalized using the pallidum CBF. ROI‐based CBFwas derived from the FreeSurfer segmentation of the T1‐weighted images using the DKT atlas^4^. ADAS‐Cog was used as a continuous measure of disease progression across multiple cognitive domains (higher scores indicate advanced disease).

The data was stratified into binned groups reflecting the ADAS‐Cog distribution and split into a 70:30 train‐test set. The meta‐ROI was constructed on the training set from 17 ROIs, selected based on their linear relationship against ADAS‐Cog. ROI‐based CBF within the meta‐ROI was used to train a regression model to predict ADAS‐Cog. Correlation was measured by Pearson’s correlation coefficient, R, on the test set.

**Result:**

We observed a correlation between meta‐ROI scores and cognitive performance of R=0.72 (Figure 1). This compared favourably with values reported in literature for PET (R=0.75) and CSF/plasma biomarkers^5^.

**Conclusion:**

The good correlation between perfusion meta‐ROI and cognitive scores supports the use of ASL‐derived biomarkers as a non‐invasive imaging alternative for predicting and monitoring disease progression.

**References**:

1. Johnson NA et al., *Radiology*. 2005;234(3):851‐859. doi:10.1148/radiol.2343040197

2. Alsop DC et al., *Magn Reson Med*. 2015;73(1):102‐116. doi:10.1002/mrm.25197

3. Chappell MA et al. Imaging Neuroscience. 2023;1:1‐16. doi:10.1162/imag_a_00041

4. Klein, Tourville. *Frontiers in neuroscience*6. 2012;171. doi:10.3389/fnins.2012.00171

5. Ossenkoppele et al, EMBO molecular medicine. 2021; doi.org/10.15252/emmm.202114398